# Displaced Distal Fibular Epiphysis in a Child: A Clinical and Radiological Evaluation of a Rare Injury Hidden From the Initial Radiological Examination

**DOI:** 10.7759/cureus.56033

**Published:** 2024-03-12

**Authors:** Nikolaos Laliotis, Panagiotis Konstantinidis, Chrysanthos Chrysanthou, Elisavet Papadopoulou, Maria Tzitiridou-Chatzopoulou, Panagiotis Dionellis

**Affiliations:** 1 Orthopaedics, Interbalkan Medical Center, Thessaloniki, GRC; 2 Orthopaedics and Traumatology, Interbalkan Medical Center, Thessaloniki, GRC; 3 Anatomy and Surgical Anatomy, Aristotle University of Thessaloniki, Thessaloniki, GRC; 4 Radiology, Interbalkan Medical Center, Thessaloniki, GRC; 5 Paediatrics and Neonatology, University of Western Macedonia, Thessaloniki, GRC; 6 Orthopaedics, Hipokrattion General Hospital, Thessaloniki, GRC

**Keywords:** lateral malleolus, salter harris distal fibula, ankle fracture children, distal fibular epiphysis, epiphysiolisthesis

## Abstract

Fractures of the lateral malleolus are common in children. Isolated lesions of the distal fibula physis commonly comprise nondisplaced or minimally displaced fractures. An isolated, completely displaced epiphysiolisthesis of the distal fibula is an extremely rare lesion. This study introduces the case of an 11-year-old boy presenting an extremely rare lesion of an isolated, completely displaced distal fibular epiphysis that was difficult to diagnose on X-ray. Initial radiographic examination of the injured ankle showed normal configuration of the tibia and fibula on AP projection, with soft tissue swelling of the lateral malleolus. On the lateral projection, the talus completely covered the distal fibular epiphysis, and particular attention was required to reveal the complete displacement of the distal fibular epiphysis. A CT scan confirmed the diagnosis of an isolated, completely displaced distal fibular epiphysiolisthesis. The patient was treated with a closed reduction and made an uneventful recovery. This report highlights the importance of accurate clinical and radiological assessment of an isolated, completely displaced, distal fibular epiphysiolisthesis.

## Introduction

An isolated, completely displaced distal fibular epiphysiolisthesis is an extremely rare injury, though fractures of the lateral malleolus are common in the pediatric population. Isolated fibular fractures commonly include nondisplaced or minimally displaced fractures. When they accompany displaced fractures of the distal tibia, displacement of the distal fibula may be found [1-3].

Physeal injuries are classified according to the Salter-Harris (SH) classification of lesions. Displacement of the distal fibular epiphysis is usually easily diagnosed by radiological examination. Undisplaced SH type 1 injuries usually present as ankle sprains. There is no obvious radiographic evidence of a fracture, except for signs of soft tissue oedema [4,5]. Two cases of isolated displaced SH type 1 fractures of the distal fibula in adolescents were previously studied. The authors described them as a variant of the ankle fracture, where the distal tibial epiphysis was near complete closure, and the forces deviated through the open physis of the fibula. Both patients were surgically treated to restore the syndesmosis and length of the fibula and to achieve a reduction of the fracture [6].

The current manuscript presents the case of an 11-year-old boy with an open growth plate of the tibia and fibula and a completely displaced type 2 SH isolated lesion of the distal fibula that was unnoticed on the initial radiographic evaluation. The AP X-ray revealed normal alignment of the tibia and fibula, with no sign of fracture. Careful inspection of the lateral X-ray revealed the displaced distal fibular epiphysis. A subsequent CT scan confirmed the diagnosis of the displaced SH 2 isolated fibular fracture. The patient had an accurate closed reduction and immobilization in a cast. A complete recovery was achieved following treatment, and a follow-up after a year showed normal growth of the distal fibula. The present manuscript aims to present the issue of hidden fractures in initial radiographic examinations and the importance of appropriate clinical and radiological examinations with the use of CT scans. Consent was obtained from the parents of the boy and the Ethical Committee of our hospital issued the approval 2168/8-2-2024.

## Case presentation

**Figure 1:**
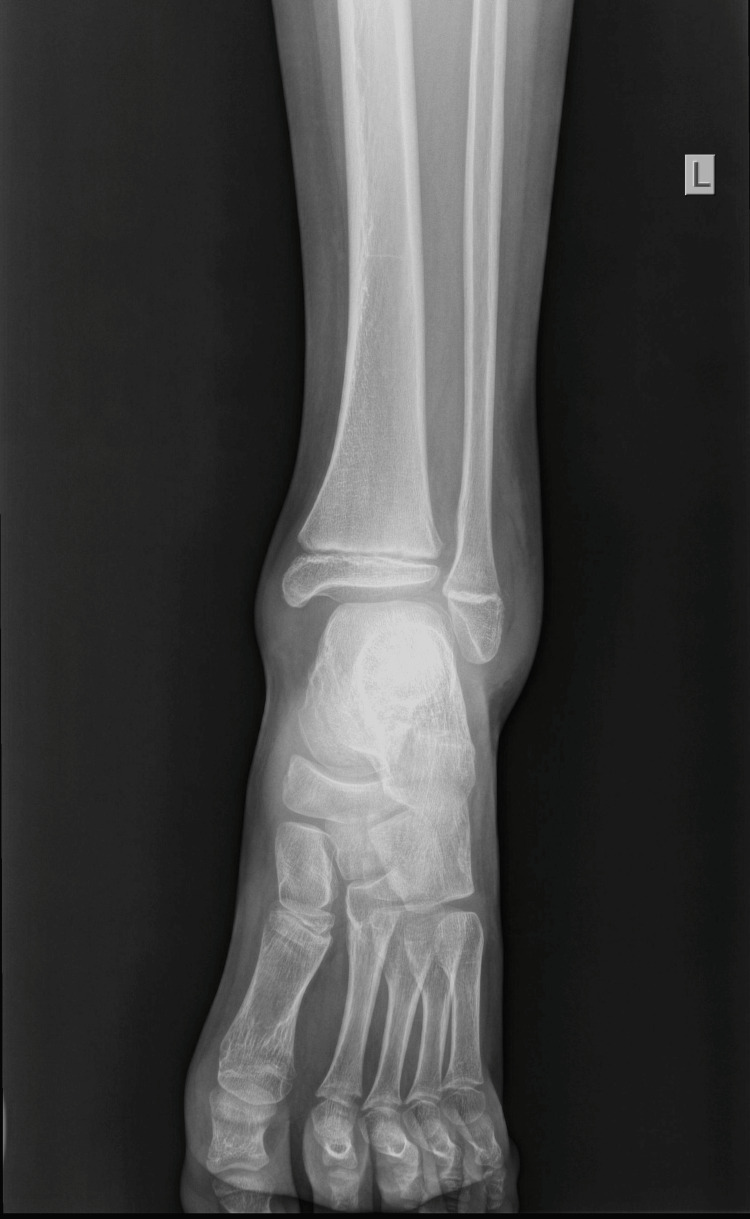
Initial X-ray AP diagnosed as an ankle sprain, with soft tissue oedema of the lateral malleolus.

**Figure 2:**
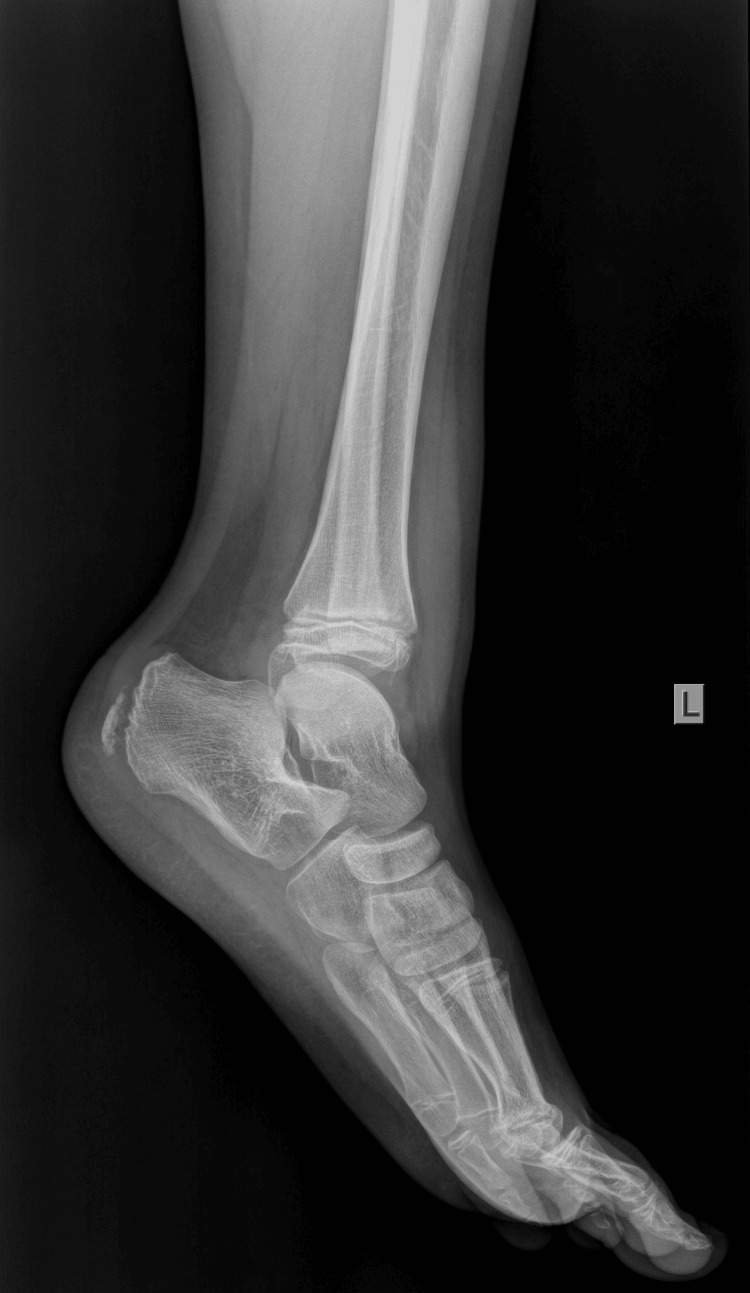
Initial X-ray lateral diagnosed as an ankle sprain, with soft tissue oedema of the lateral malleolus.

An 11-year-old boy sustained an injury to his left ankle while playing football. He reported that he felt as though his foot was trapped on the field while running. He expressed severe pain and was unable to stand on the injured foot. He was transferred to the emergency department of the local hospital, and an AP and lateral X-ray of the affected ankle was requested. The initial diagnosis indicated a severe ankle sprain without signs of a fracture; soft tissue oedema was also observed (Figures 1-2).

**Figure 3:**
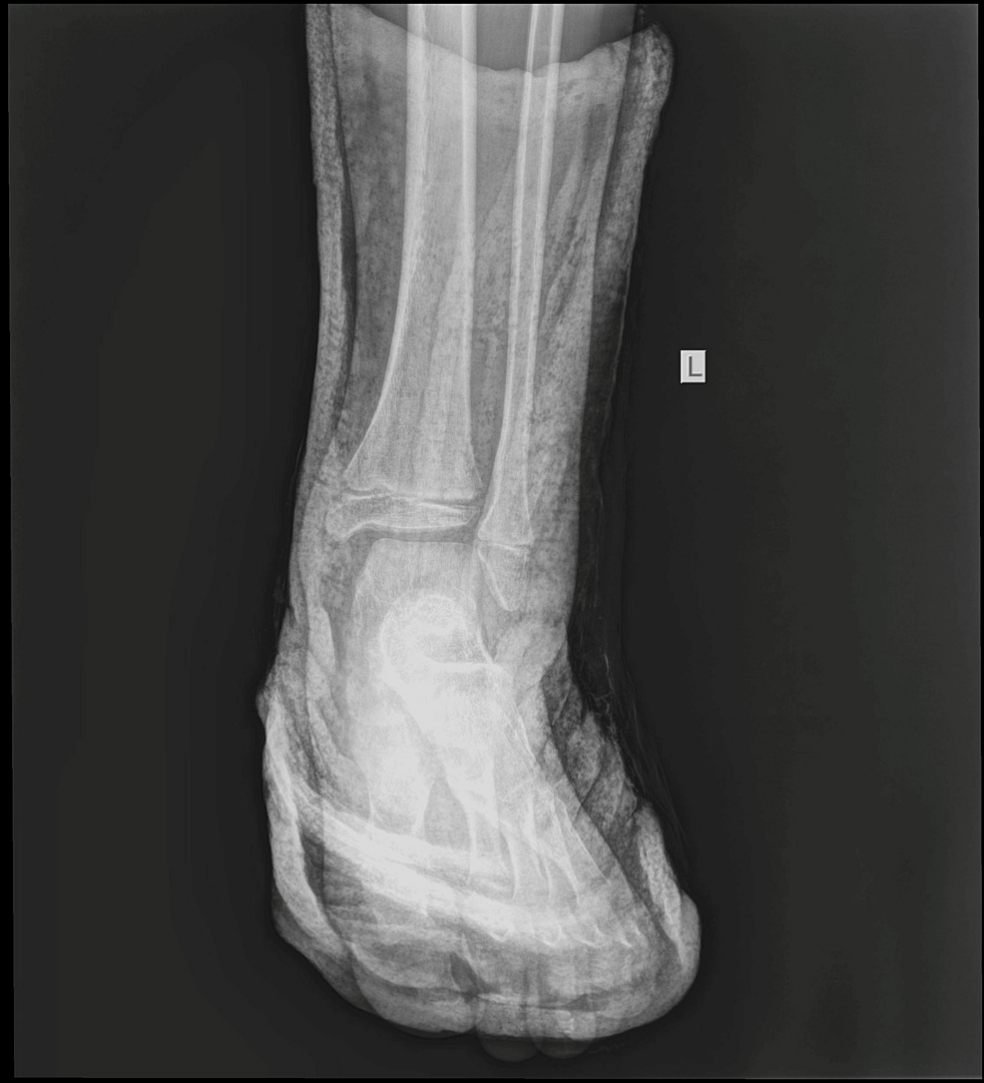
X-ray AP, after immobilization in a cast

**Figure 4:**
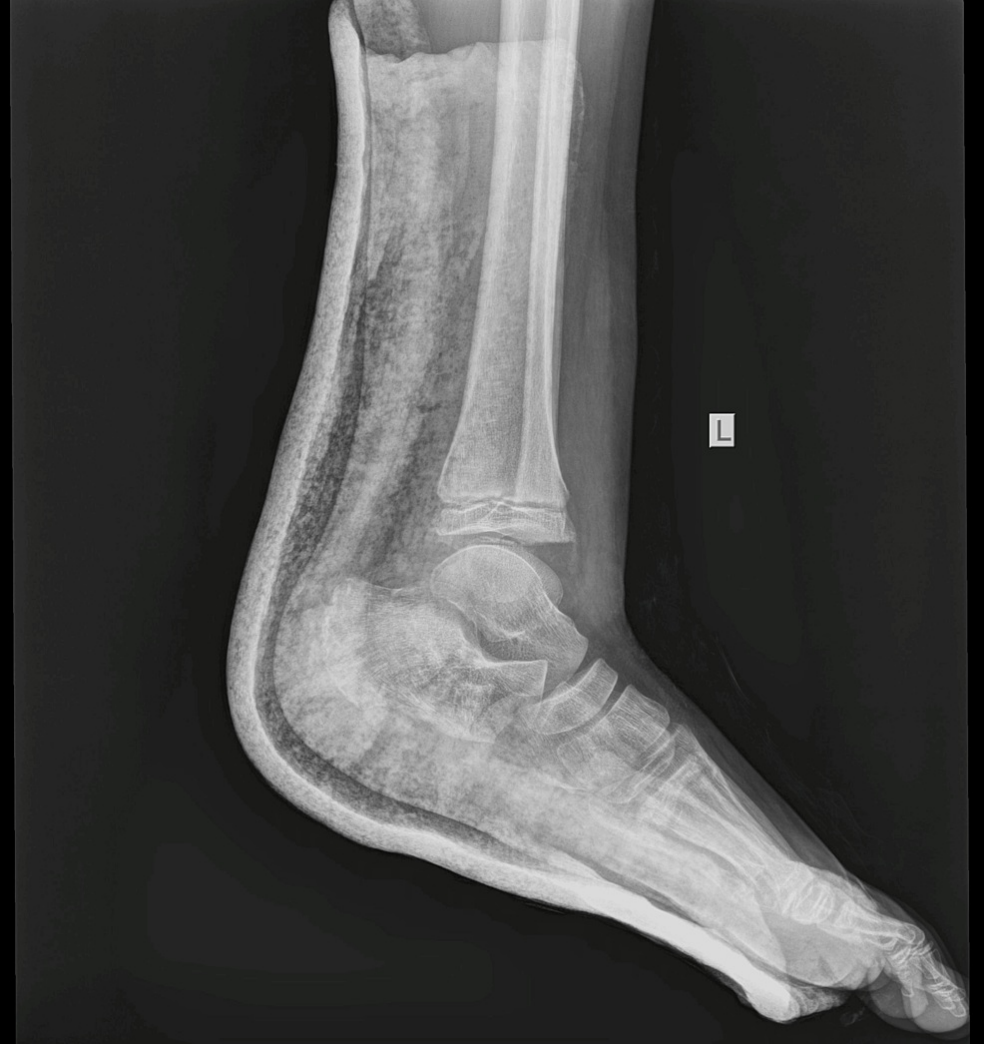
X-ray lateral, after cast immobilization

The foot was immobilized in a cast in an almost neutral position. A radiological examination with the cast was repeated to confirm normal ankle configuration (Figures 3-4).

**Figure 5:**
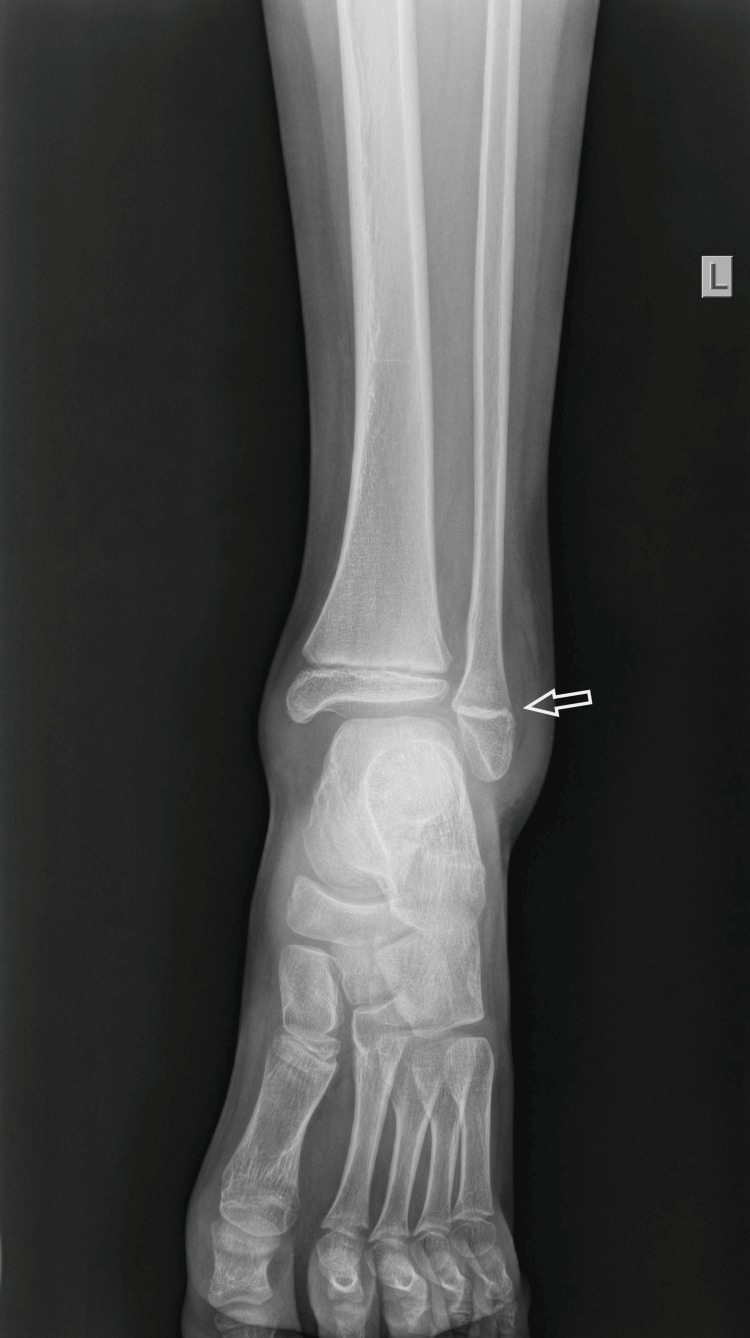
Reviewing the initial AP X-ray of the ankle joint, with the arrow pointing to the reduced width of the distal physis of the fibula.

**Figure 6:**
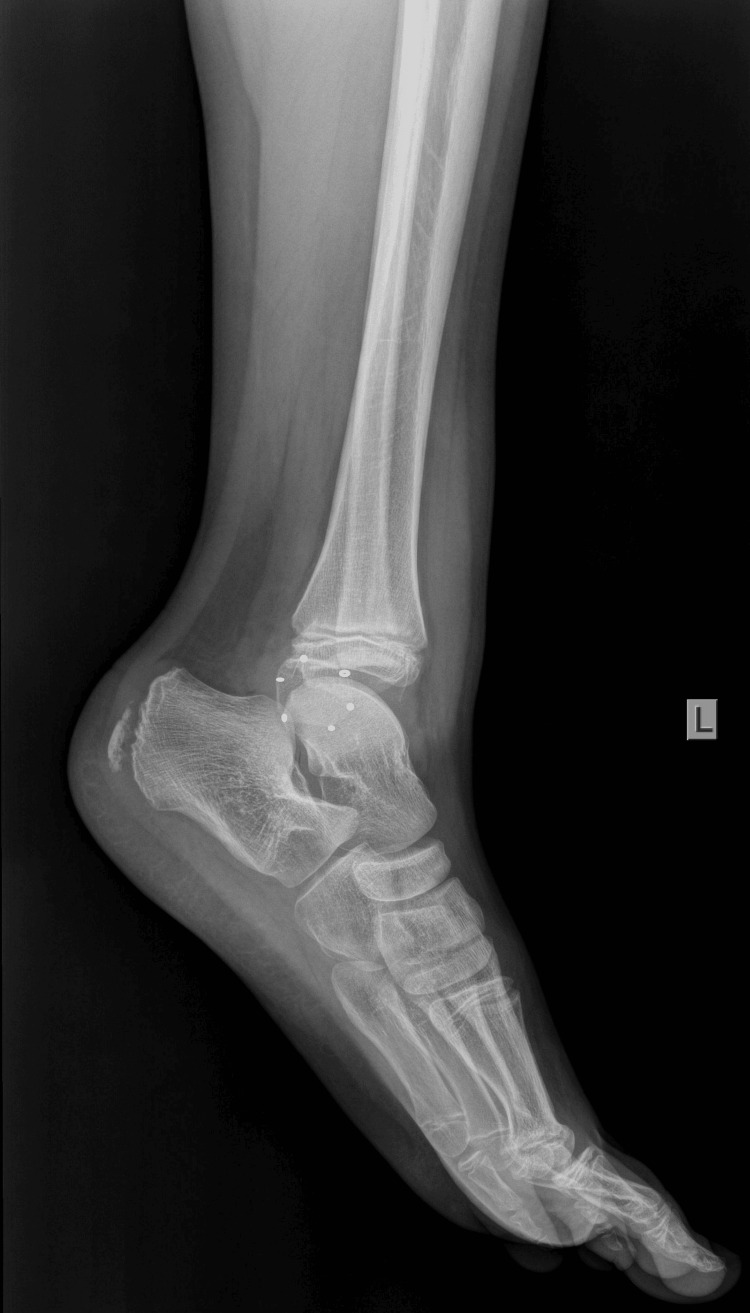
Reviewing the lateral X-ray with the dotted line outlining the distal fibular epiphysis that is completely displaced in relation to the fibular metaphysis.

The boy returned to his house with the advice to keep his foot elevated and to apply ice above the cast. The next morning, the boy was referred to our pediatric orthopedic department for another evaluation. He was experiencing a great deal of pain. On removal of the soft cast (plaster of Paris - POP), there was oedema on the lateral side of the ankle at the distal end of the fibula. On reviewing the initial radiological evaluation of the AP projection, the ankle joint appeared normal, without any displacement of the tibia and fibula. However, on careful inspection, the width of the distal fibular physis was observed to be reduced, compared to the open physis of the tibia. On the lateral X-ray, the distal fibular epiphysis was hardly outlined as it was projecting over the talus. Nonetheless, it was possible to confirm the disruption of the entire fibula, which was superimposed on the tibia, from the distal fibular epiphysis. The diagnosis of a completely displaced distal fibular epiphysis was confirmed (Figures 5-6).

**Figure 7:**
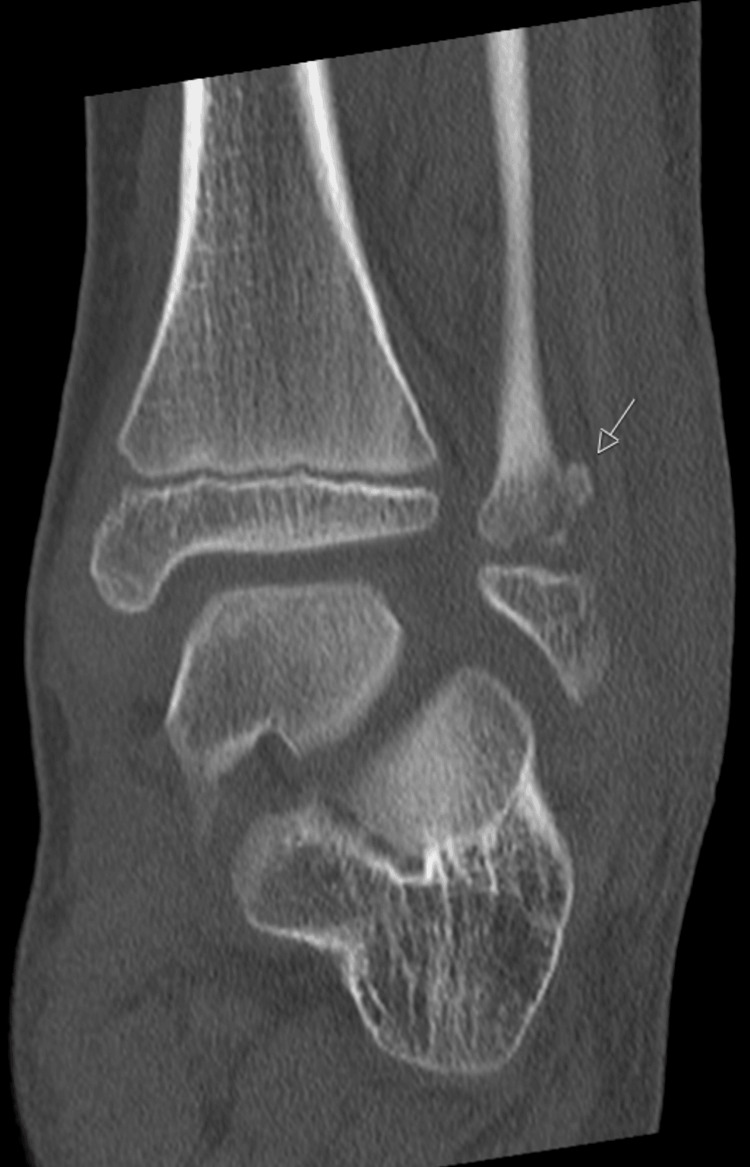
An AP projection of the CT scan, with the arrow pointing the metaphyseal fragment of the distal fibular epiphysiolisthesis.

**Figure 8:**
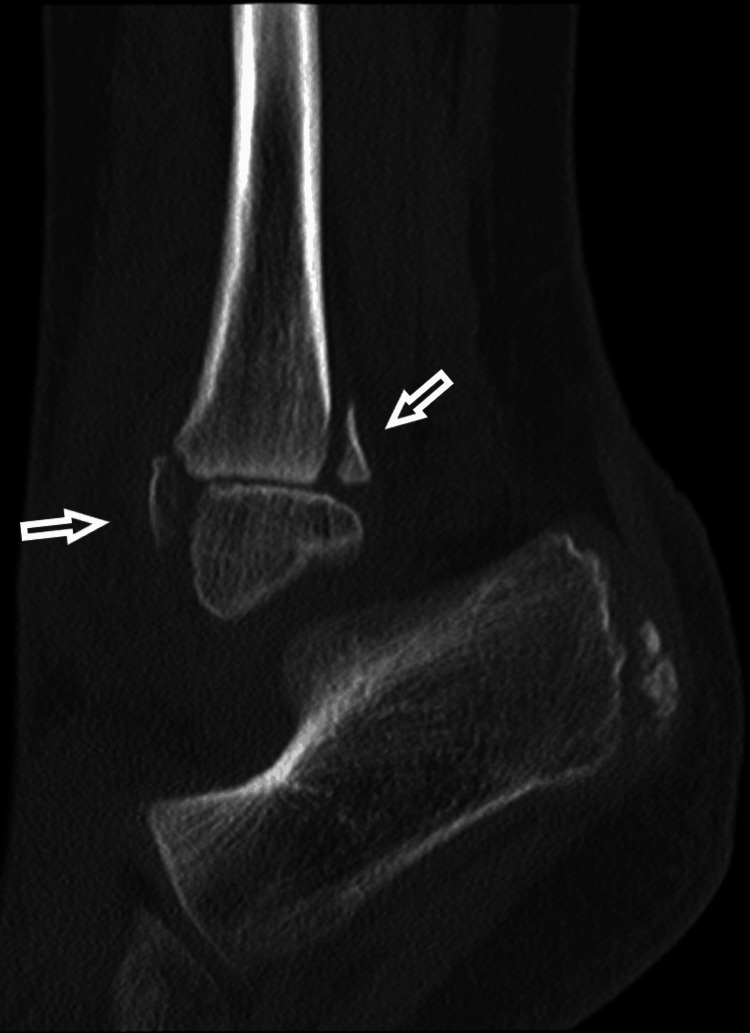
A lateral projection of the CT scan, with the arrows pointing to the metaphyseal fragments of the distal fibular epiphysiolisthesis.

**Figure 9:**
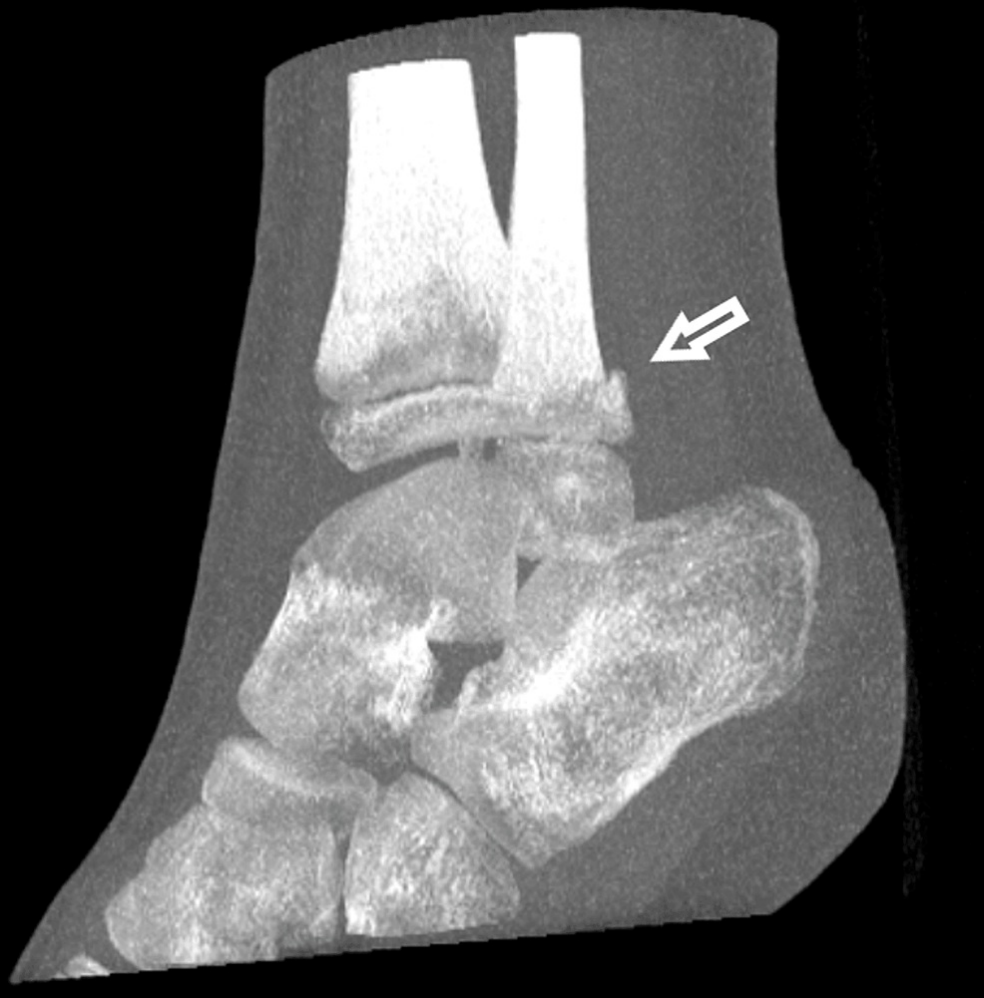
A 3D picture with the arrow at the distal fibular epiphysiolisthesis

A CT scan was performed, further confirming the diagnosis of epiphysiolisthesis of the distal fibula and showing adequate reduction. There was a small fragment of the metaphysis of the fibula, and the tibial growth plate was intact (Figures 7-9).

**Figure 10:**
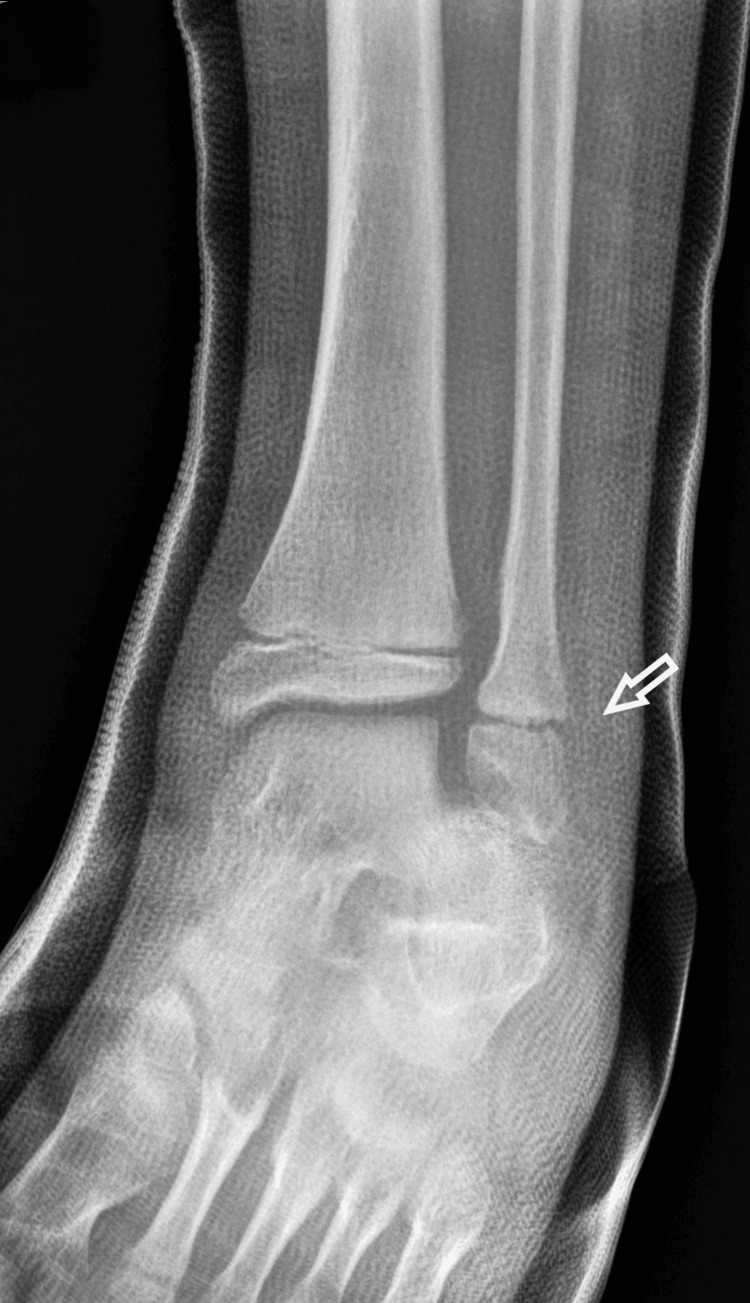
AP X-ray, after reduction of the fracture and immobilization in a cast. The arrow is pointing the normal alignment of the distal fibular epiphysis.

**Figure 11:**
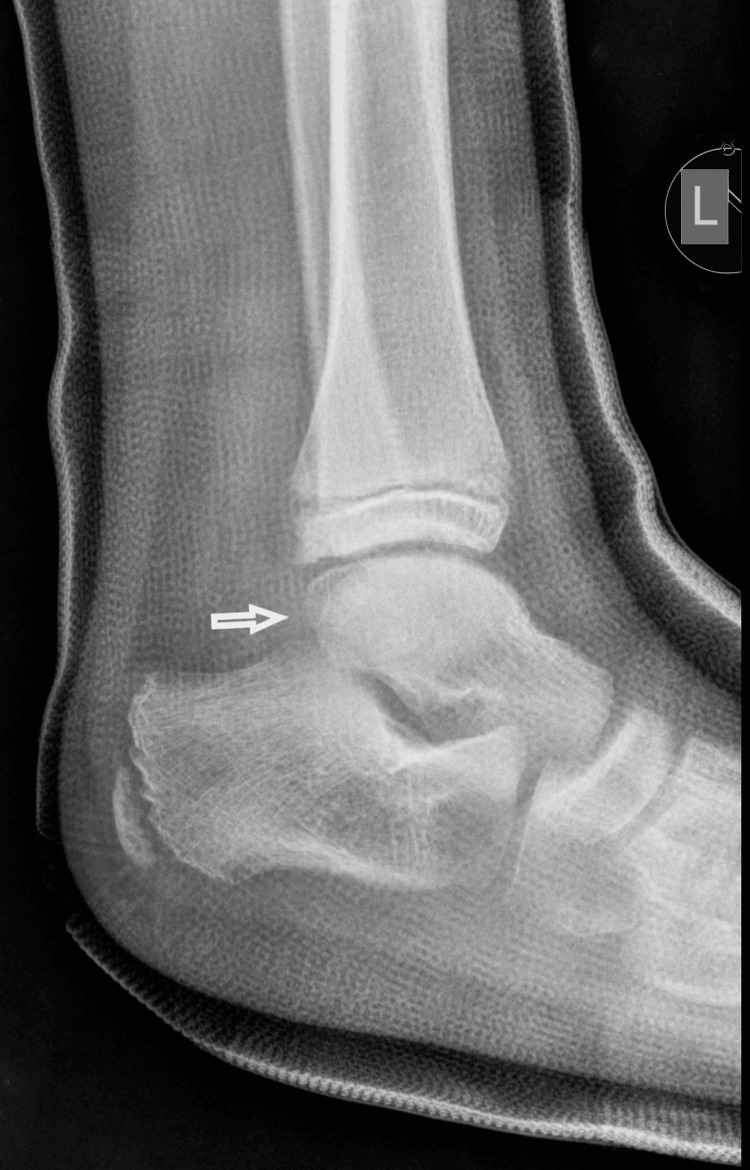
Lateral X-ray, after reduction of the fracture and immobilization in a cast. The arrow is pointing the normal alignment of the distal fibular epiphysis.

We gradually further reduced the fracture under gentle manipulation as the boy was very cooperative by reversing the ankle in a mild varus and neutral position. We immobilized the patient in a cast below the knee. The patient expressed relief of pain and discomfort. Immediate radiological examination confirmed the complete reduction, with restoration of the width of the distal fibular physis on the AP projection and normal position of the distal fibular epiphysis in the lateral projection, in line with the proximal fibula (Figures 10-11).

**Figure 12:**
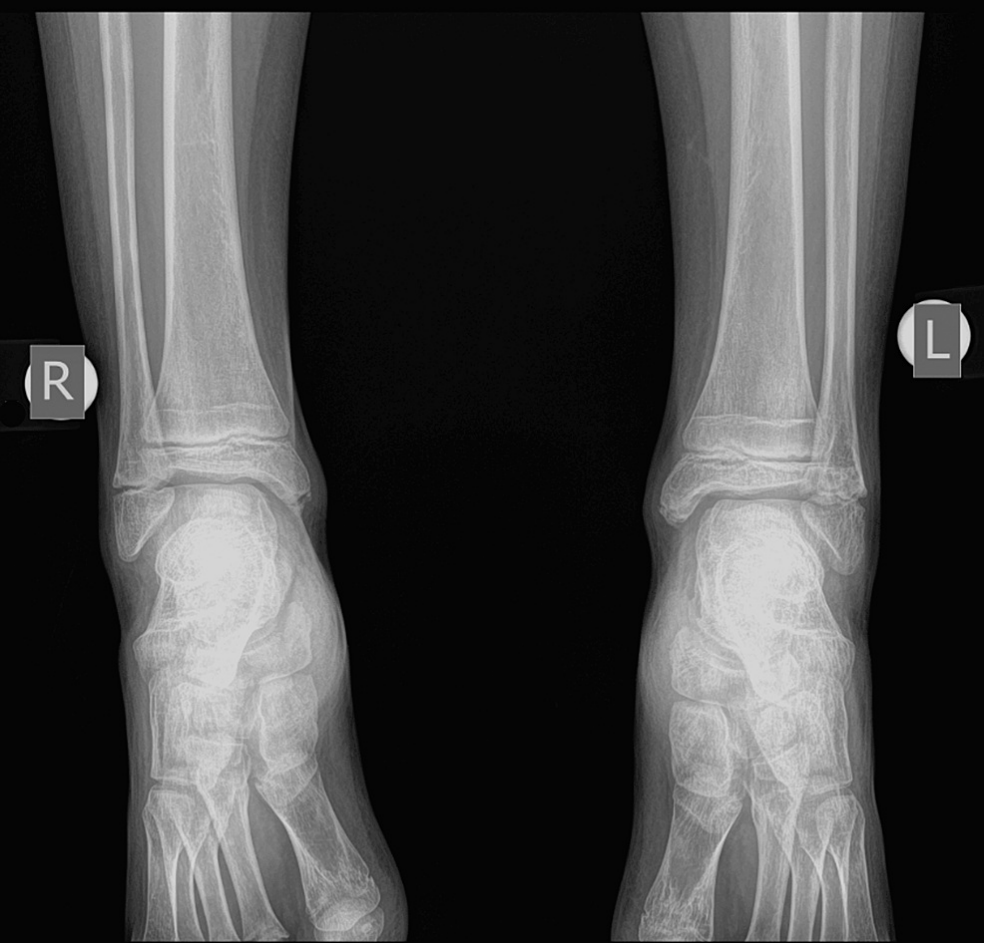
A comparative X-ray of both ankle joints, one year after injury, with normal growth of the left distal fibula.

**Figure 13:**
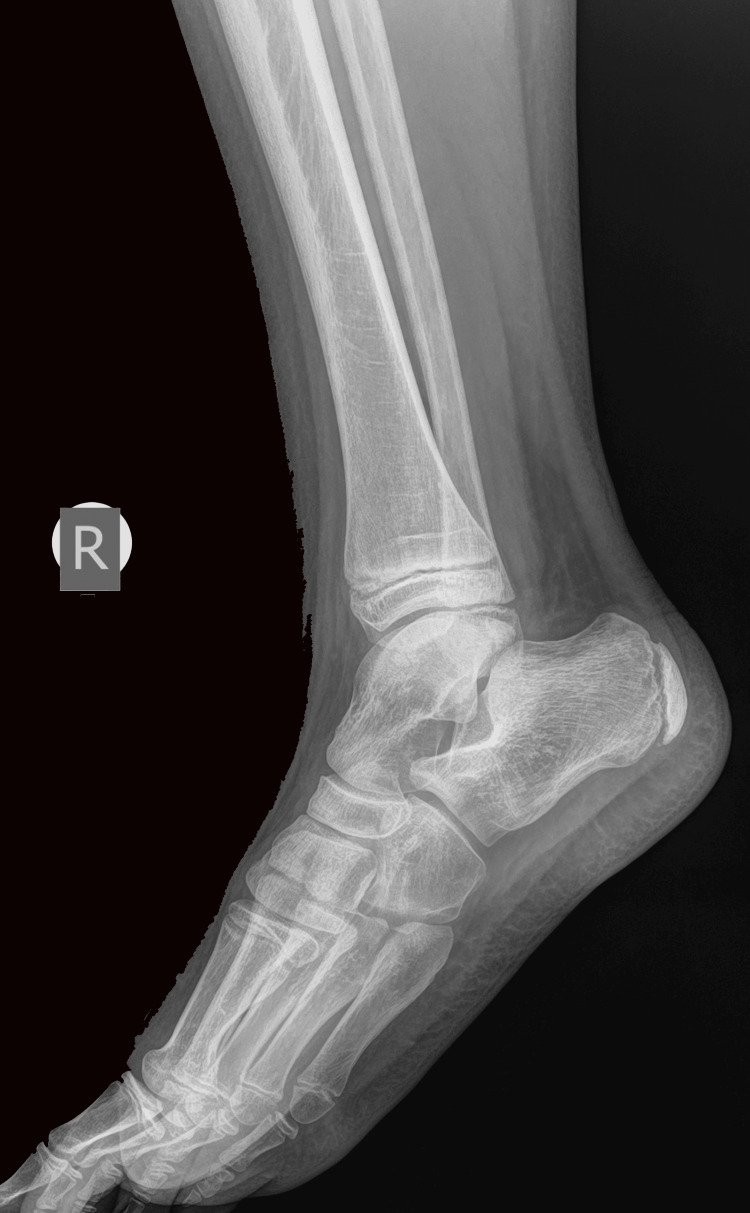
Lateral X-ray of the right ankle

**Figure 14:**
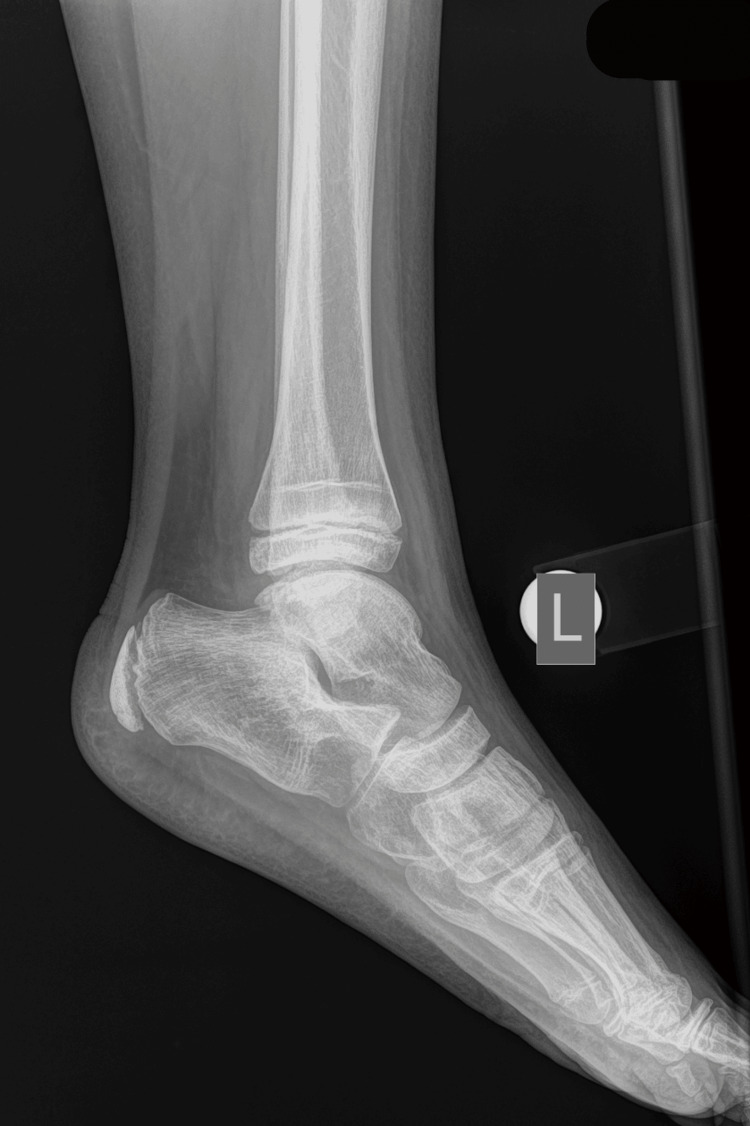
Lateral X-ray of the left injured ankle

The fracture was immobilized for four weeks. The patient was examined after one week and two weeks with X-rays to confirm the preservation of the reduction. The cast was removed after one month. He had a period of another month with crutches for partial weight bearing before returning to normal walking. He returned to sports activities three months after the initial injury. He was last reviewed one year after the initial injury, with a complete range of motion (ROM) of the ankle. The radiological examination confirmed normal growth of the distal fibula and normal configuration of the ankle in a comparative X-ray of both ankle joints (Figures 12-14).

## Discussion

Lateral ankle fractures are the most common sports injuries in children and young adolescents. Fractures that include the growth plate are described according to the SH classification. Fractures of the distal fibular growth plate are commonly type 1, with undisplaced epiphysis, presenting clinically localised tenderness and oedema [3,4]. Undisplaced fractures have a normal radiological appearance of the fibula, presenting only soft tissue swelling on X-rays. Physical examination was reported to be unreliable in accurately locating the distal fibular epiphysis [7].

Children presenting with localised tenderness over the distal fibular physis after an injury and without radiological evidence of a fracture are commonly diagnosed with undisplaced SH1 fractures of the distal fibula (SH1DF). Hofli et al. have used MRI to examine children aged 8-12 years who were clinically and radiologically diagnosed with SH1 fractures. They have examined 38 patients out of 391 who were diagnosed at the emergency department with ankle sprain. None of them showed evidence of fracture of the physis. They had ligamentous lesions, joint effusion or bone oedema [8]. Boutis et al. reported the same result in 18 patients, clinically suspected to have SH1DF, when none of them had evidence of an SH1 fracture on MRI examination [9].

Displaced SH types 1 or 2 fractures of the distal fibula are easily diagnosed on X-ray evaluation. Korsch and Adolefsen presented two patients with isolated displaced SH type 1 that were easily diagnosed on X-ray, with displacement of the distal fibular epiphysis. Both patients were treated with an open reduction and stabilization [6]. In our patient, the AP projection appeared with a normal position of the distal fibular epiphysis. It is important to properly clinically examine the patient to reveal that increased localised tenderness and soft tissue oedema are correlated with a severe injury and not a simple sprain. The difference in the width of the physis is an important clue for the proper diagnosis of the fibular epiphysiolisthesis. On the lateral view, it is difficult to follow the configuration of the distal fibula that is superimposed on the tibia.

Closed reduction of epiphysiolisthesis is easily achieved unless there is periosteal entrapment that may require open reduction and fixation in unstable cases. Lugeder et al. reported a rare injury of a SH 4 distal fibular fracture with disruption of the tibiofibular ligament. The diagnosis was confirmed with a CT scan. Treatment was performed with open reduction and fixation with K wire [10]. CT scan can be used to identify and accurately classify fractures of the distal tibial epiphysis. Karlikowski et al. studied a total of 75 patients aged 7-17 years with physeal fractures and classified them using SH assessment. Lateral malleolus fracture was found to coexist with fractures of the tibial physis which was the main reference lesion. Only four patients had an isolated type 1 fibular fracture, and another four had fractures of the tip of the lateral malleolus [2]. SH type 3 fractures are occasionally difficult to diagnose from normal subfibular ossicles. A subfibular ossicle is present in 1% of the population as a result of failure of fusion of the ossification centre or after trauma [11]. Sugi et al. reported that radiographic assessment can help to determine the presence of fracture versus an os subfibulare [12]. Tillaux or triplane fractures must be examined with CT to clarify the extent of the fracture and the amount of displacement. The use of CT may revise the initial classification after a plain X-ray and is helpful in accurately planning either closed or open reduction and appropriate fixation. Using CT, we can estimate the size of a metaphyseal fragment in SH 2 fracture, called the Thurston-Holland fragment. The size of the metaphyseal fragment is not an important factor for premature physeal closure (PPC) in fractures of the distal tibial, according to Turgut et al. [13]. Most important are the amount of displacement, the increased number of attempts for closed reduction and the penetration of the physis from K wires. In our patient, the CT scan revealed a small metaphyseal fragment of the fibula, measuring less than 10% of the diameter of the physis. Giacobazzi et al. recently reported a nondisplaced SH 3 fracture of the distal fibula while radiologically, using a CT scan, examining a Tillaux fracture of the distal tibial epiphysis. They described the uncommon combination of concomitant epiphysiolisthesis, both of tibia and fibula. They performed closed reduction and pinning of the tibial epiphysis using screw fixation [14]. PPC is a severe complication following an epiphyseal fracture of the distal tibia. This is not reported for lesions of the distal fibular physis [2,3,15]. Karlikowski and Sulko reported that the periosteum interposition in fractures of the distal tibial physis is regarded as an important factor in avoiding PPC [2]. De Santis et al. reported that the fundamental factor for the prognosis of distal tibial and fibular epiphyseal fractures in children was the mechanism of trauma and the damage to the physis [3]. PPC is not reported in growth plate lesions of the distal fibula [6]. One case of a lesion of a seven-year-old boy, who had missed part of the epiphysis after being struck by a vehicle, was previously reported [16]. Our patient had an uneventful recovery, with normal development of the distal fibula, and he is now back to full sports activities.

## Conclusions

We present the initially hidden lesion of the complete displacement of the distal fibula epiphysis to underline the importance of combining clinical evaluation and radiological findings. CT examination is helpful for lesions of the ankle joint in children and can confirm the accurate reduction of the epiphysiolisthesis. This is important for normal development of the ankle joint.
